# Who Participates in the Great ShakeOut? Why Audience Segmentation Is the Future of Disaster Preparedness Campaigns

**DOI:** 10.3390/ijerph14111407

**Published:** 2017-11-17

**Authors:** Rachel M. Adams, Beth Karlin, David P. Eisenman, Johanna Blakley, Deborah Glik

**Affiliations:** 1 Center for Public Health and Disasters, Fielding School of Public Health, University of California at Los Angeles, Los Angeles, CA 90095, USA; rachelad@ucla.edu (R.M.A.); deisenman@mednet.ucla.edu (D.P.E.); 2 The Norman Lear Center, Annenberg School for Communication and Journalism, University of Southern California, Los Angeles, CA 90089, USA; bethkarlin@gmail.com (B.K.); blakley@usc.edu (J.B.); 3 Division of General Internal Medicine and Health Services Research, David Geffen School of Medicine, University of California at Los Angeles, Los Angeles, CA 90095, USA

**Keywords:** earthquake preparedness, emergency drills, community resilience, audience segmentation

## Abstract

*Background*: In 2008, the Southern California Earthquake Center in collaboration with the U.S. Geological Survey Earthquake Hazards Program launched the first annual Great ShakeOut, the largest earthquake preparedness drill in the history of the United States. *Materials and Methods*: We collected online survey data from 2052 campaign registrants to assess how people participated, whether audience segments shared behavioral patterns, and whether these segments were associated with five social cognitive factors targeted by the ShakeOut campaign. *Results*: Participants clustered into four behavioral patterns. The Minimal cluster had low participation in all activities (range: 0–39% participation). The Basic Drill cluster only participated in the drop, cover and hold drill (100% participation). The Community-Oriented cluster, involved in the drill (100%) and other interpersonal activities including attending disaster planning meetings (74%), was positively associated with interpersonal communication (β = 0.169), self-efficacy (β = 0.118), outcome efficacy (β = 0.110), and knowledge about disaster preparedness (β = 0.151). The Interactive and Games cluster, which participated in the drill (79%) and two online earthquake preparedness games (53% and 75%), was positively associated with all five social cognitive factors studied. *Conclusions*: Our results support audience segmentation approaches to engaging the public, which address the strengths and weaknesses of different segments. Offering games may help “gamers” gain competencies required to prepare for disasters. Targeting the highly active Community-Oriented cluster for leadership roles could help build community resilience by encouraging others to become more involved in disaster planning. We propose that the days of single, national education campaigns without local variation should end.

## 1. Introduction

### 1.1. Background

In Southern California, despite the many years of education and campaigns about earthquake preparedness and response, studies continually suggest that people are not fully prepared for disasters nor have they completely processed what actions are most important to take during an earthquake [1,2,3]. In a 2009 survey, Kano and colleagues found that less than 40% of Southern California respondents made a family disaster plan and less than 10% participated in neighborhood disaster planning [1]. Some respondents also remained misinformed about what to do during an earthquake; believing the doorway is the safest place inside a building was the most commonly held myth [1]. In another survey conducted in Los Angeles, Eisenman and colleagues found that only 40% of respondents reported having a family communication plan, with lower rates among ethnic minority groups, persons with lower income, and persons with chronic illnesses [2]. The prevalence of disaster supplies among households in Los Angeles was slightly higher (48%) [2]; however, these types of statistics tend to be inflated, as people often only have some but not all the recommended supplies [3].

The public’s lack of overall engagement in preparedness and response activities has provided impetus for more creative and engaging ways to motivate the public to prepare. In response to this demand, the USGS Earthquake Hazards Program collaborated with the Southern California Earthquake Center at the University of Southern California to spearhead the first Great ShakeOut drill. This novel community-based drill incorporates well-known drivers of behavior change. A central component of the drill involved learning through behavioral rehearsal. At the most basic level, participants physically practiced the “drop, cover and hold” actions, while others also rehearsed parts of their worksite’s evacuation, triage, and reunification plans. According to Bandura’s Social Cognitive Theory, rehearsing a behavior improves one’s ability to learn it, as practice allows them to refine the behavior as a skill [4]. Rehearsing the drill can thus help participants build the appropriate competencies needed to respond to a disaster. Additionally, the drill was conducted in a group setting at schools, worksites, businesses, and other organizations and thus encouraged participants to learn the behavior within a social context. Social Cognitive Theory posits that people learn from one another via observation and social modeling, which can then enhance their self-efficacy for engaging in a behavior [5]. Self-efficacy, which is a measure of one’s perceived ability to succeed in a specific action [5,6], is a recognized correlate of household and community disaster preparedness [7,8,9]. Learning within a social context can also reinforce norms and attitudes surrounding the behavior, encouraging outcome expectations [6]. Positive expectations of a behavior are also important correlates of disaster preparedness [10,11]. Finally, the recruitment of a wide range of businesses, schools, worksites and community organizations to participate in the drill allowed for multiple family members and friends to take part in different locations at the same time. This aimed to promote “milling” or further discussion of the drill and other disaster planning, which has been shown to enhance engagement in disaster preparedness behaviors [12,13].

In addition to the drill, a range of communication initiatives were launched in the months leading up to it to raise public awareness about the drill and earthquake preparedness in general. For instance, there was a dedicated website where participants could access the following items: a narrative booklet, video and audio recording describing a 7.8 earthquake scenario linked to California’s San Andreas fault [13,14,15]; a drill manual that included information about developing an organizational and household disaster plan; graphic materials that could be printed to promote participation in the drill; and links to two educational video games called Beat the Quake and After Shock. Other initiatives included the development of ShakeOut groups on social media websites, community outreach meetings, and a two-day tabletop exercise. These interpersonal activities aimed to enhance community engagement in disaster preparedness, drawing on the notion that shifts in social norms accompany changes at the individual level. Advertisements for the drill were additionally disseminated via broadcast radio as well as posters, billboards and electronic billboards displayed throughout the Los Angeles metropolitan area [13,14]. The “drop, cover and hold” message of these advertisements utilized a prescriptive approach for earthquake preparedness that aimed to elicit a very specific set of behaviors. These tactics are rooted in disaster risk communication research, as well as health communication and social marketing campaigns more generally, which suggests that people best process messages that are simple, consistent and actionable [16,17,18,19,20,21].

### 1.2. Research Considerations and the Present Study

In recent years, there has been a global push for greater community involvement in disaster preparedness efforts in order to build resilience. For instance, the U.S. Federal Emergency Management Agency outlines a strategic framework for implementing “whole community” approaches to engage the public, including understanding the community’s needs, strengthening existing community relationships, and engaging and empowering all parts of the community [22]. The U.S. Centers for Disease Control and Prevention’s Public Health Emergency Preparedness grants also require that recipients meet specific performance measures related to community capacity building, such as partnering with community stakeholders and involving them in planning efforts [23]. Globally, there are several frameworks that support this approach, such as the International Federation of Red Cross’ Framework for Community Resilience [24] and United Nations Office for Disaster Risk Reduction’s Sendai Framework, which emphasizes the need to empower and involve local communities in the development of disaster risk reduction policies, plans, and legislation [25]. Given the heightened interest in preparedness programs that involve and engage the community, we are presented with a valuable opportunity to better inform these efforts by evaluating the comprehensive community-based ShakeOut drill and campaign.

Following the launch of the first Great Southern California ShakeOut in 2008, we surveyed those who registered for the campaign about their participation in the drill and other campaign activities. We also collected data on social cognitive factors known to enhance engagement in disaster preparedness that were targeted by the campaign’s best practices, including interpersonal communication, personal responsibility, self-efficacy, outcome efficacy, and knowledge about earthquake preparedness. Using these survey data, the current study aims to assess (1) what types of campaign activities did survey participants enact; (2) whether there are discernable patterns of behavior in which audience segments cluster; and (3) whether these audience segments are associated with the social cognitive factors targeted by the ShakeOut campaign. Previous research supports the value of audience segmentation, as people who share certain behavior patterns and beliefs may benefit from tailored messaging and outreach [9,26]. By identifying and analyzing these audience segments, our findings can help inform how large population- or community-based drills disseminate to the public and how those who participate best respond to these efforts.

## 2. Materials and Methods

### 2.1. Study and Survey Design

An online cross-sectional survey was conducted five months after the launch of the Great Southern California ShakeOut drill among a sample of registered participants to learn about participation in ShakeOut activities and social cognitive factors that may have been influenced by the campaign and drill. We selected and analyzed the survey measures listed below.

*Demographic and personal characteristics.* The following four demographic variables were collected: gender, age, race/ethnicity, and income. We also included a measure of personal experience with a disaster as this is a well-known correlate of participating in disaster preparedness behaviors [21].

*ShakeOut participation.* Respondents were asked if they participated in eight ShakeOut activities. These activities consisted of (1) participating in the “drop, cover and hold” drill on 13 November at 10 a.m.; (2) practicing a disaster plan; (3) helping others prepare for the ShakeOut; (4) participating in a meeting in their workplace or school about preparing for earthquakes; (5) joining a MySpace ShakeOut group; (6) joining a Facebook ShakeOut group; (7) playing the Beat the Quake game on the ShakeOut Website; and (8) signing up to play AfterShock, which became available after the launch ShakeOut drill.

*Social cognitive factors*. We measured interpersonal communication by asking respondents if they had spoken with family or friends during the past 30 days about earthquakes in general, as well as about their disaster communication plan, earthquake kits, preparing their home for an earthquake, their community disaster plan, and having extra cash on hand. Responses were coded binary (No = 0, Yes = 1). For personal responsibility, respondents ranked the level of importance that they feel personally responsible about being prepared for an earthquake. Responses ranged from 1 (not important) to 5 (extremely important). Self-efficacy was measured by asking respondents three questions about whether they felt capable of preparing for a major earthquake by making the right decisions about (1) water, (2) food, and (3) making a disaster communication plan. Answers ranged from 1 (strongly disagree) to 5 (strongly agree). We assessed outcome efficacy using six questions about how preparing for an earthquake might help if a big earthquake happens. The respondents were asked how much they agreed with the following statements: (1) having a disaster kit may help me; (2) if furniture and appliances are secured, my home may be safer; (3) working with local community may make it easier for my household; (4) storing cash may help my household; (5) calling an out-of-area contact may help me after an earthquake; and (6) having a plan for meeting up with a family may be helpful. Answers ranged from 1 (strongly disagree) to 5 (strongly agree). Finally, knowledge was measured via a 22-item quiz, which asked respondents to choose the correct actions to do in the event of an earthquake. These items were broken up into five sections that asked about what they should do during an earthquake if they were inside a building, outside a building, in bed, or driving, as well as what they should do after the earthquake.

### 2.2. Study Area

The first Great ShakeOut campaign and drill took place in the greater Los Angeles region, which is the second most populous urban area in the United States. It is also one of the most diverse regions in the United States in terms of socioeconomic status, culture, religion, household type, and the economy [27]. Los Angeles is ranked the top city in North and South America for the number of people potentially affected by a natural disaster due to the two major earthquake faults it sits on, as well as other hazards [28]. The U.S. Geological Survey estimates that there is a 99.7% chance that Southern California will experience an earthquake of 6.7 or larger during the next 30 years [29].

### 2.3. Sampling Size and Data Collection

Our goal was to interview as many individuals who registered for the ShakeOut campaign as possible in order to obtain a diverse sample of participants. No a priori power analysis was conducted to calculate sample size. The survey was sent to the full registry of individuals who signed up to participate in the ShakeOut campaign and drill, which was a total of 18,411 people.

### 2.4. Data Collection

Invitations to the online survey were emailed on 22 April 2009. The survey design was based on Dillman’s Tailored Design Method [30] in which progress indicators, multiple screens, and a simple layout were used to maximize survey completion. The survey took approximately 15 min to complete. To incentivize participation, all survey participants were entered into a raffle with a chance to win a US$100 gift card. No reminders about the survey were sent and it was kept open for 20 days (closing on 11 May 2009).

### 2.5. Data Analysis

First, we ran descriptive statistics for the demographic and ShakeOut participation variables. For gender and race, we calculated percentages within each pre-specified category. To make direct comparisons with Census data statistics, we calculated means for age and income by imputing the mean within each response category (e.g., 30–39 years converted to 34.5). For ShakeOut participation, we calculated the percentages of people participating in each ShakeOut activity.

Next, we ran a factor analysis to identify underlying categories of behaviors from the different ShakeOut activities. We used principal component extraction, Varimax rotation and Kaiser Normalization. We used the Scree test to identify the number of factors. Next, we ran a two-step cluster analysis so that we could derive behavioral pattern clusters using the behavior categories identified through factor analysis. With all behaviors included, no clusters emerged, so we removed the factors with less than 5% participation and conducted two-step cluster analysis with the following three input variables: (1) drop, cover and hold; (2) interpersonal behaviors; and, (3) games. We then ran a series of chi-square tests to determine if there were significant variations in the distribution of demographic variables across the various ShakeOut participation clusters.

Finally, we ran multiple linear regression models to examine whether demographic variables, personal experience with a disaster, and the ShakeOut behavior clusters were significantly associated with each social cognitive factor (interpersonal communication, personal responsibility, self-efficacy, outcome efficacy, and knowledge). The demographic variables included in the models were gender, age, race/ethnicity, and income. For social cognitive variables, we created composite scales for multi-item variables by calculating the averages across each response. Each of these variables was treated as a continuous dependent variable in each regression model.

### 2.6. Ethical Consideration

The study protocol was reviewed and approved by an institutional review board of the University of Southern California. Study: Evaluation of an Earthquake-Preparedness Campaign (UP-08-00347).

## 3. Results

The survey was started by 2390 people with 2052 completing the survey (13% response rate)**.** Table 1 presents summary statistics of the survey sample’s demographic characteristics compared to 2010 California Census data [31]. In comparison to the Census data, our sample was older, possessed higher incomes, and consisted of a larger number of females and white participants. The sample, although not representative, proved sufficiently diverse to address the research questions proposed by the study.

The frequencies for participation in each ShakeOut activity are located in Table 2. The most frequent activity was the drop, cover and hold drill (71%) and the least frequent was joining MySpace (0%) and Facebook (3%). About a third practiced a disaster plan, helped others prepare for the ShakeOut, or attended a ShakeOut meeting. Approximately 10% played each of the games. The factor analysis performed on the eight ShakeOut activities yielded four factors, which accounted for 64.8% of the total variance (see Table 2). Participation in the drop, cover and hold drill was one factor. The second factor, which we called the interpersonal factor, included practicing a disaster plan, helping others prepare for the ShakeOut; and participating in a ShakeOut meeting. A third factor, games, consisted of participating in the Beat the Quake game and registering for the After Shock game. The fourth factor, social media, included joining the Facebook and MySpace groups.

Four clusters emerged from our analysis, which are depicted in Table 3. About a quarter of respondents clustered into the Minimal cluster, which possessed low participation in all activities, with only a minority participating in interpersonal behaviors. The greatest percentage of people (38.5%) was in the Basic Drill cluster, which consisted of 100% participation in practicing the drop, cover and hold drill, with minimal participation in interpersonal behaviors and no participation in games. The third cluster (20.3% of participants), which we called the Community-Oriented cluster, did not participate in the ShakeOut games, but possessed a very high involvement in practicing a disaster plan, helping others prepare for the Shakeout, and participating in a ShakeOut meeting. This cluster also had 100% participation in the drop, cover and hold drill. The fourth and smallest cluster (15.6% of participants), the Interactive and Games cluster, possessed lower participation in the basic drop, cover and hold drill than the previous two clusters, about half of the people participating in each of the interpersonal activities, and was the only cluster where many individuals were playing or registered for the two online ShakeOut games.

Frequency distributions of demographic characteristics for each cluster and the chi-square results are provided in Table 3. The following variables possessed statistically significant variation across the different clusters at 0.05 alpha level: gender, white race, Latino ethnicity, ages 18–29, 30–39, 40–49, and 60 years and older.

Results from the regression analyses are presented in Table 4. The predictor variables in the regression models explained from 4 to 12% of the variance in outcomes, ranging from 4.4% for knowledge to 12.4% for interpersonal communication. Among demographic variables, being female was positively associated with outcome efficacy and knowledge about earthquakes. In comparison to white participants, African Americans and Asians had lower self-efficacy, whereas Latinos had higher outcome efficacy. All three racial/ethnic minority groups also had lower earthquake preparedness knowledge in comparison to white participants. Participants in the highest income category ($75,000 or more) exhibited significantly higher interpersonal communication and knowledge compared to those in the lowest income group ($25,000 or less). Age and personal experience with a disaster also had strong effects on all outcomes except knowledge, with older participants and those with previous experience associated with greater interpersonal communication, personal responsibility, self-efficacy, and outcome efficacy.

In comparison to the Minimal cluster, being in the Basic Drill cluster was only significantly associated with greater knowledge about what to do during an earthquake. Belonging to the Community-Oriented cluster was also associated with higher knowledge, as well as enhanced self-efficacy, outcome efficacy, and interpersonal communication. Being in the Interactive and Games cluster was positively associated with all five social cognitive outcomes. However, the strength of the associations was not as strong as those seen for the Community-Oriented cluster, which had particularly high coefficients, especially for interpersonal communication (β = 0.169).

## 4. Discussion

The Great Southern California ShakeOut represents an evolution in risk communication and planning efforts to prepare communities for catastrophic disasters. When developing the drill and campaign leading up to it, risk communication was considered within a larger social context and followed a skill-based training approach. The audience was not simply encouraged to plan for an earthquake by having household supplies or a family communication plan, but was also trained to practice specific behaviors in direct response to an earthquake. The main behavior practiced in this exercise was to “drop, cover and hold” when an earthquake occurs. Not surprisingly, the drop, cover and hold drill was the most frequently cited activity among survey respondents. Additionally, the audience was encouraged to connect with others when planning for and participating in the drill. About a third of the participants engaged in interpersonal activities by attending a meeting where preparing for earthquakes was discussed, practiced a disaster plan such as a work evacuation or family reunification plan, and helped to prepare for the ShakeOut drill. Participants also connected with others via media-based interactions offered through the earthquake games and social media groups; however, these activities were less popular than the interpersonal activities. In particular, joining the campaign’s Facebook and MySpace groups was the least cited activity by a substantial margin. Despite social media’s potential to enhance social connectedness [32], it appears that our sample of participants was more amenable to in-person interaction than these newer community engagement techniques. This is perhaps a reflection of the time frame of the survey when social media use was less prevalent [33].

Using the three most popular campaign behaviors, we were able to cluster the audience segments into distinct behavioral patterns. We found that the majority of respondents fell into the Basic Drill cluster, which participated in the drop, cover and hold drill, but was less interested in the other campaign activities. This segment represents a group that is likely open to a one-time group exercise, but is less engaged in the more involved and interactive campaign activities. In contrast, those who belonged to the Community-Oriented cluster represent a group that is demonstrably active in community planning efforts by also attending meetings, practicing additional plans, and helping others. This group had a greater proportion of Latinos, a demographic group that has previously been shown to respond better to preparedness interventions that utilize discussion-based learning and social networks [34]. The distinction between the Basic Drill and Community-Oriented clusters suggests that the campaign could benefit from better audience segmentation approaches that are targeted to the strengths and weaknesses of different groups. For instance, future iterations of the ShakeOut campaign and other similar interventions could offer additional opportunities for individuals to host meetings and tabletop exercises so that more engaged participants can recruit and encourage others in their social network to get more involved.

This study supports findings from a prior cluster analysis conducted by Adams and Eisenman on a different dataset of Southern California residents [9]. In that study, we also discerned the existence of a very active cluster in the community who engages in community capacity and skill building. The study found that one-third of the very active cluster attended a community meeting or volunteered to help their community become disaster prepared. It also found that Latinos were more likely to be in the community-oriented cluster after controlling for other covariates. More importantly, both analyses found strong associations between self-efficacy and the most active, community-oriented clusters, supporting the finding that self-efficacy goes beyond influencing household preparedness by contributing to broader community resilience. Thus, we now have two studies demonstrating that community disaster resilience activities can be divided into discrete domains and behavior patterns with associated variations in social cognitive characteristics. Once again, these results tell us that emergency managers and public health practitioners need to exploit the lessons of audience segmentation further by providing the most engaged persons with opportunities and resources to contribute to local community resilience building. By encouraging these individuals to transfer their knowledge and skills to others in the community, emergency managers can gain additional resources and support needed to move beyond telling the public to stockpile supplies—the default practice in disaster preparedness messaging. Other researchers have recommended similarly, notably Abramson’s herd preparedness strategy, which emphasizes that we can increase population preparedness by providing highly involved individuals with tools to develop formal response structures [26,35].

Despite the fact that we surveyed a highly motivated sample that had registered for the ShakeOut drill, 26% of respondents still fell into the Minimal cluster, a group that did not actually participate in the drop, cover and hold drill. While disappointing, this finding was not entirely surprising, as it is well documented in the literature that most individuals do not follow the recommended behaviors for disaster preparedness despite the existence of education and campaigns [13,36]. This audience segment likely possesses specific barriers to participating in a community-based drill, making them a particularly hard population to reach. One way to better target this audience segment would be to develop a social network recruitment strategy for the ShakeOut campaign where highly engaged participants are assigned formal recruitment roles at their participating organizations. Those in the Community-Oriented cluster would be perfect for these roles, as the vast majority (89%) of them already indicated that they helped others prepare for the ShakeOut drill. By encouraging these more active individuals to talk to their colleagues about the drill and gain leadership support to host different activities, we hypothesize that they could help individuals who are interested in the drill but who are not yet ready or able to commit.

The fourth cluster, Interactive and Games, possessed a unique pattern of behavior. While approximately half of them participated in interpersonal activities, they expressed greater interest in games as a way to get more involved in the campaign beyond the basic drill. Three-quarters of this group played the Beat the Quake game posted on the ShakeOut website and more than half even signed up for the After Shock game before it was made available. The existence of this “gamer” group supports the inclusion of virtual games as an additional interactive platform to engage different population segments. In fact, results from the regression models demonstrate the value of learning and practicing disaster preparedness activities through virtual games. After controlling for demographic characteristics and personal experience with disasters, the Interactive and Games cluster was positively associated with all five social cognitive factors that were targeted by the ShakeOut campaign. If we compare these findings to those for the Basic Drill cluster, which was only positively associated with earthquake knowledge, it appears as though playing games further contributed to interpersonal communication about earthquake preparedness, as well as perceptions of personal responsibility, self-efficacy, and outcome efficacy regarding these behaviors. These results suggest that the act of playing games can promote further discussion of preparedness among family and friends. Practicing preparedness behaviors in a virtual setting where an earthquake is simulated may also enhance feelings about their personal responsibility and ability to prepare for an earthquake, as well as perceptions that these behaviors can successfully protect against the destructive consequences of these disasters.

Results from the regression models also demonstrate the benefit of belonging to the Community-Oriented cluster. This group was positively associated with interpersonal communication, self-efficacy, outcome efficacy, and knowledge about earthquake preparedness, which are all factors known to engage the public in disaster preparedness and response [8,36,37]. Interestingly, this cluster was not associated with a higher rating of perceived personal responsibility for preparedness, which may reflect their more collective outlook for disaster planning. They also possessed a particularly strong association with interpersonal communication about earthquakes, disaster communication plans, preparing their home for earthquakes, emergency supplies, and their community disaster plan. Discussion of these topics was a major priority of the campaign because communities are more resilient if friends, families, neighbors, and colleagues know how to assist one another in emergency situations [38]. With community resilience acting as the guiding framework for a number of global disaster preparedness programs and strategies [22,23,24,25,39,40], there has been greater emphasis on activities that empower and engage communities to contribute to collective disaster planning and community preparedness. For instance, the Sendai Framework for Disaster Risk Reduction asks that we promote a culture of disaster prevention, resilience and responsible citizenship by encouraging stakeholders to be engaged in regional education and awareness campaigns [25]. Our results support the value of preparedness programs that offer these more involved, interpersonal activities, such as community meetings, as there are segments of the population who want to participate and who benefit from their involvement. Targeting these individuals for additional participation and leadership opportunities may also improve preparedness among the entire population. The U.S. Centers for Disease Control and Prevention’s Office of Public Health Preparedness and Response has also emphasized the need to prioritize research that evaluates the effectiveness of novel and persuasive risk communication approaches for changing knowledge, attitudes and practices [41]. We suggest that emergency managers conduct audience segmentation surveys prior to program development and implementation to identify and target highly involved audience segments. Evaluating these novel techniques could then reveal whether audience segmentation approaches are more effective than traditional education approaches and ultimately guide future efforts to influence disaster preparedness.

### Limitations

Despite our numerous findings, our study is bounded by certain limitations. First, our respondents were a highly self-selected pool among those already registered for the ShakeOut drill. Our results are therefore not generalizable to everyone who participated in the campaign. Self-selection of more engaged participants may have also reduced some of the variation in the outcomes across different segments of the population. Additionally, having the chance to win a $100 gift card as an incentive to participate may have influenced who self-selected to participate in the study and may have led to inappropriate completion of the study questionnaire for the sake of the incentive. Second, while we attempted to encourage survey participation through the $100 incentive, we still had a very low response rate which likely contributed to non-response bias. We thus recognize that our findings are not generalizable to all registrants of the campaign. Third, although our survey intended to capture information about social cognitive characteristics that may have been influenced by participation in the ShakeOut drill and campaign, our data were cross-sectional in nature. While the study design encourages linear temporality by surveying respondents several months after the campaign, the cross-sectional design still only allows us to make inferences on the associations between variables and not causation. Fourth, our multiple regression models had fairly low *R*^2^ values, indicating that the independent variables did not explain a large proportion of the total variation in the models. The inclusion of additional unmeasured variables may have improved model fit. Finally, our study examined individuals who participated in the first ShakeOut drill that was launched in 2008. While this provides us with insight into who was registered for the first campaign as well as how to improve participation in the Great ShakeOut and other community-based drills, further research is needed to examine how newer changes, such as greater social media presence and the introduction of new interactive games and virtual reality experiences, enhance participation and overall disaster preparedness.

## 5. Conclusions

While the majority of respondents only participated in the drop, cover and hold drill, we identified a more motivated, community-oriented audience segment who we believe should be targeted for leadership roles in order to get others more involved as well as improving social connectedness, which is a key feature of resilience. Additionally, playing online earthquake games contributed to important social cognitive factors for disaster preparedness, including personal responsibility, self-efficacy, and outcome efficacy. We recommend that preparedness drills and programs include more interactive in-person and virtual activities in order to target different segments of the population and enhance overall community resilience. Furthermore, we recommend emergency managers conduct audience segmentation surveys prior to program development and implementation to elucidate who are their distinct audience segments and use these results to provide a panoply of opportunities targeted to the right audience. We believe that the days of single, national education campaigns without local variation should end.

## Figures and Tables

**Table 1:** Demographic Characteristics of the Sample (*N* = 2052) Compared to 2010 CA Census Data.

	Study Sample	Census Data
Gender *	Female 66.1%	Female 50.3%
Race/ethnicity *	White 74.4%	White 57.6%
	Hispanic/Latino 14.3%	Hispanic/Latino 37.6%
	Asian/Pacific Islander 7.4%	Asian/Pacific Islander 13.4%
	African American 3.8%	African American 6.2%
Age *	48.8 Years	35.2 Years
Income *	$65,309	$59,540

* Sample and census significantly different based on independent *t*-test (*p* < 0.01).

**Table 2:** Factors derived from participation in ShakeOut activities (*N* = 2052).

		Factor Analysis			
	Frequency of Participation (%)	Drop, Cover, Hold	Interpersonal	Games	Social Media
	Loading				
Drop, cover and hold during drill	71%	**0.902**	0.090	0.038	−0.014
Practice a Disaster Plan	39%	−0.311	**0.728**	0.088	−0.005
Help others prepare for Shakeout	39%	0.117	**0.781**	0.062	0.015
Participate in a meeting	33%	0.311	**0.645**	0.022	−0.036
After Shock game	8%	−0.001	0.024	**0.841**	0.012
Beat the Quake game	12%	0.042	0.108	**0.814**	0.025
Join Facebook	3%	0.116	−0.057	0.124	**0.750**
Join MySpace	0.3%	−0.136	0.042	−0.087	**0.763**
Explained variance		13.2%	19.8%	17.6%	14.2%

Note: Values in bold indicate which items load to each factor.

**Table 3:** Frequency distribution of ShakeOut activities and demographic variables in each derived cluster (*N* = 2052).

	Minimal Cluster	Basic Drill Cluster	Community-Oriented Cluster	Interactive and Games Cluster	
Participation	26%	38%	20%	16%	
Basic					
Drop, cover, hold	0%	100%	100%	79%	
Interpersonal					
Practice Plan	39%	15%	76%	52%	
Help others	29%	15%	89%	53%	
Attend Meeting	23%	15%	74%	43%	
Game					
Aftershock game	0%	0%	0%	53%	
Beat the Quake	0%	0%	0%	75%	
Demographics					Chi-Square (*p* value)
Female Gender	63%	71%	67%	70%	11.29 ^a^ (0.010)
Race/Ethnicity					
White	80%	69%	77%	76%	19.42 ^a^ (<0.001)
African American	2%	3%	5%	4%	5.68 (0.128)
Latino	9%	14%	18%	15%	16.10 ^a^ (0.001)
Asian/Pacific Islander	8%	7%	8%	5%	3.41(0.333)
Income					
<$25,000	16%	15%	11%	12%	4.60 (0.204)
$25,000–$49,999	19%	21%	22%	22%	1.54 (0.673)
$50,000–$74,999	25%	26%	25%	25%	0.07 (0.995)
>$75,000	41%	38%	42%	40%	1.76 (0.624)
Age					
18–29	7%	11%	7%	9%	9.22 ^a^ (0.027)
30–39	12%	15%	11%	18%	9.10 ^a^ (0.028)
40–49	21%	25%	29%	26%	8.45 ^a^ (0.038)
50–59	29%	28%	31%	32%	2.78 (0.426)
60+	31%	21%	23%	14%	31.95 ^a^ (<0.001)

^a^ *p* < 0.05.

**Table 4:** Linear regression of demographics and ShakeOut participation clusters on social cognitive factors (*N* = 2052).

	Interpersonal Communication	Personal Responsibility	Self-Efficacy	Outcome Efficacy	Knowledge
	Standardized Regression Coefficient (β)				
Gender					
Male (reference)	--	--	--	--	--
Female	−0.038	0.030	0.008	0.107 ^a^	0.054 ^a^
Race/Ethnicity					
White (Reference)	--	--	--	--	--
African American	−0.003	−0.038	−0.058 ^a^	0.014	−0.090 ^a^
Latino	0.026	0.011	0.024	0.101 ^a^	−0.065 ^a^
Asian/Pacific Islander	0.005	−0.025	−0.070 ^a^	0.022	−0.067 ^a^
Income					
<$25,000 (Reference)	--	--	--	--	--
$25,000–$49,999	0.047	0.043	0.038	−0.008	0.043
$50,000–$74,999	0.054	0.044	0.075	−0.023	0.043
>$75,000	0.100 ^a^	0.059	0.056	−0.036	0.086 ^a^
Age					
18–29 (Reference)	--	--	--	--	--
30–39	0.067	0.060	0.104 ^a^	−0.026	0.028
40–49	0.137 ^a^	0.137 ^a^	0.106 ^a^	0.081	0.077
50–59	0.242 ^a^	0.192 ^a^	0.204 ^a^	0.200 ^a^	0.106 ^a^
60+	0.227 ^a^	0.205 ^a^	0.189 ^a^	0.222 ^a^	0.080
Personal Disaster Experience	0.171 ^a^	0.140 ^a^	0.098 ^a^	0.176 ^a^	−0.012
ShakeOut Behavior Cluster					
Minimal (Reference)	--	--	--	--	--
Basic Drill	−0.044	−0.021	0.008	0.012	0.068 ^a^
Community-Oriented	0.169 ^a^	0.034	0.118 ^a^	0.110 ^a^	0.151 ^a^
Interactive and Games	0.117 ^a^	0.079 ^a^	0.086 ^a^	0.069 ^a^	0.144 ^a^
*Adjusted R*^2^	0.124	0.051	0.056	0.094	0.044

^a^ *p* < 0.05.
